# Prevalence and Zoonotic Potential of *Giardia intestinalis* in Dogs of the Central Region of Mexico

**DOI:** 10.3390/ani9060325

**Published:** 2019-06-06

**Authors:** Elsa M. Godínez-Galaz, Nerina P. Veyna-Salazar, Andrea M. Olvera-Ramírez, Feliciano Milián-Suazo, Claudia A. Perea-Razo, Rodolfo Bernal-Reynaga, Germinal J. Cantó-Alarcón

**Affiliations:** 1 Doctorado en Ciencias Biológicas, Facultad de Ciencias Naturales, Universidad Autónoma de Querétaro, Avenida de las Ciencias S/N Juriquilla, Delegación Santa Rosa Jáuregui, Querétaro C.P. 76230, Mexico; egody03@hotmail.com (E.M.G.-G.); claudia.a.perea@gmail.com (C.A.P.-R.); 2 Maestría en Salud y Producción Animal Sustentable, Facultad de Ciencias Naturales, Universidad Autónoma de Querétaro, Avenida de las Ciencias S/N Juriquilla, Delegación Santa Rosa Jáuregui, Querétaro C.P. 76230, Mexico; nerina.veyna@uaq.mx; 3 Cuerpo Académico Salud Animal y Microbiología Ambiental, Facultad de Ciencias Naturales, Universidad Autónoma de Querétaro, Avenida de las Ciencias S/N Juriquilla, Delegación Santa Rosa Jáuregui, Querétaro C.P. 76230, Mexico; andrea.olvera@uaq.mx (A.M.O.-R.); feliciano.milian@uaq.mx (F.M.-S.); 4 Cuerpo Académico Consolidado de Salud Pública, Unidad de Investigaciones en Salud Pública “Dra. Kaethe Willms”, Facultad de Ciencias Químico Biológicas, Universidad Autónoma de Sinaloa, Ave. de las Américas y Blvd. Universitarios, Ciudad Universitaria, Culiacán, Sinaloa C.P. 80100, Mexico; rodolfobernal@uas.edu.mx

**Keywords:** *Giardia intestinalis*, zoonotic assemblages, cross-transmission

## Abstract

**Simple Summary:**

*Giardia intestinalis* is a parasite that causes disease in different species, including humans and dogs. It is prevalent worldwide and disease in humans is mainly the consequence of poor hygiene habits and close interaction with infected animals. Because cross infections can be seen between humans and dogs, it is critically important to know which genotypes affect each species, useful for determining if dogs are the source of infection for humans in a particular setting. The aim of this study was to determine if dogs in the central region of Mexico play a significant role in human infections from this same area. Sampling of dog feces was performed in animal shelters, dog breeding establishments, animal control facilities and homes. Direct coproparasitoscopic diagnosis was performed; PCR and RFLP analysis were used to obtain genotypes. Results from all of the positive samples show that the genotypes from dogs matched *G. intestinalis* assemblage A, which does cause infection and disease in humans. These findings highlight the fact that infection in dogs by *G. intestinalis* needs to be controlled in order to stop transmission amongst the dog population, and, most importantly, to prevent human contagion.

**Abstract:**

*Giardia intestinalis* is a protozoan of worldwide distribution capable of infecting a large number of species, including humans and domestic animals. Dogs represent a risk to public health due to cross-infections by the zoonotic assemblages. However, there is little information concerning the prevalence and frequency of this parasite and its assemblages in dogs of the central region of Mexico, thus this study aimed to contribute to this matter. A total of 402 feces samples from dogs of different settings (shelter, breeding establishments, domestic and stray) were obtained and direct coproparasitoscopic examination by flotation revealed a prevalence of 25%. PCR was performed for amplification of the *β-Giardin* gene, to which 24 samples were positive. Assemblages were obtained through RFLP analysis, using enzymes *Hae III* to obtain the main genotypes (A–G), and *Hha I* to subtype assemblage A. All 24 samples were genotyped as assemblage A, with 83% as AI and 17% as AII. Thus, these findings confirm that dogs in the central region of Mexico are a risk for zoonotic transmission of this parasite, emphasizing the importance of a much needed control of the disease in this species.

## 1. Introduction

*Giardia intestinalis* is the flagellated protozoan more frequently involved in gastrointestinal diseases in a wide range of species, including humans. It belongs to the class Zoomastigophorea, order Diplimonadida, and is of worldwide distribution [1,2,3]. The parasite forms cysts, which are then excreted in the stool; cysts are capable of surviving in the environment, and fecal–oral transmission begins with the ingestion of the cysts, and at least 10 cysts need to be ingested to cause disease [4,5,6]. This parasitosis, as is the case with other gastrointestinal diseases, is generally the result of poor hygiene in the food-preparation process [7]. Worldwide, it is estimated that 200 million people present giardiasis each year, with a mortality rate estimated at 500,000 people, mainly during the early stages of the disease, with children as the most vulnerable population, followed by pregnant women and people with immunodeficiency disorders [3,5,8].

*G. intestinalis* is divided into eight genetic groups or assemblages (A–H) that are morphologically very similar, but genetically different. These genetic differences can be analyzed through molecular techniques such as polymerase chain reaction (PCR), restriction fragment length polymorphism (RFLP) and sequencing of the genes *gdh*, *tpi*, and *β-Giardin* [2,9,10]. The zoonotic assemblages are A and B, which mostly affect humans; in dogs, aside from A and B, there is also C and D, which are considered to be species-specific. However, there have been reports of infection in humans by assemblage C [11,12,13].

Infected individuals may be asymptomatic or undergo severe illness that can be acute or chronic, including symptoms such as fetid diarrhea, nausea, headache, fever, pain and abdominal distension [14]. The presentation of the disease depends on the type of assemblage causing the infection: assemblage A is associated to acute infections, while B is chronic, and, unlike assemblage A, it has been reported in the majority of symptomatic cases [15,16,17]. Additionally, there are other factors that contribute to the type of illness that develops, mainly the presence of other parasites or bacteria, and the nutritional and immunological states of the host [11,15].

In Mexico, the frequency of this parasitosis in humans is 19%, affecting mainly toddlers and school-aged children [8]. In dogs, worldwide prevalence is estimated at 15.2% [18], while in Mexico it is higher than 42% [19]. The main assemblages reported in humans and dogs in Mexico, in Mexico City and Sinaloa, are AI and AII [20,21]. This strongly suggests a significant problem for public health in Mexico with regards to zoonotic transmission, in addition to socio-cultural factors and sanitary conditions [22]. However, Feng and Xiao [10] mentioned that the predominant assemblages found in dogs are the species-specific C and D. Regardless, researchers from Spain and China found infection in humans by assemblage C, thus, considering that it is species-specific, this strongly confirms the participation of dogs in the transmission of this parasitosis to humans [13,23]. Therefore, dogs represent a high risk for public health due to the high prevalence of zoonotic assemblages [19,21,24]. Unfortunately, there is a lack of information on the prevalence of this parasite for the central region of Mexico, as well as for the involvement of the different dog populations in the area. Thus, the objective of this study was to obtain the prevalence of *G. intestinalis* in dogs from this region, as well as the frequency at which the zoonotic assemblages participate, and determine the risk to the human population due to cross-infections.

## 2. Materials and Methods

This project was approved by the Bioethics Committee of the Natural Sciences Department of the Autonomous University of Queretaro under registry number 42FCN2016.

### 2.1. Location, Experimental Design and Sampling

This was a transversal study performed in the city of Santiago de Querétaro from February to December 2018. A total of 402 feces samples were obtained from different populations of dogs. For each sample, the data recorded were age (<6 months or >6 months), gender (male or female), consistency of the feces (firm or mounding) and type of population, which was divided into four main criteria: breeding establishment, defined as a group of dogs of a specific breed that are in a confined space with means of reproduction of that specific breed; shelter, animals without an owner/home or abandoned that are confined in a single place and in close contact with other dogs; strays, animals that live on the streets; and domestic, animals that live in homes as pets.

### 2.2. Coproparasitoscopic Diagnosis

To determine the prevalence of the parasite, a direct parasitoscopic analysis through a simple flotation technique [25] was performed. First, 2 g of feces were mixed into 10 mL of distilled water and the mixture was homogenized and filtered. Second, the filtered solution was placed in 15 mL conical tubes and centrifuged at 350× *g* for 5 min. The supernatant was decanted and the precipitate was mixed into 10 mL of distilled water, and the mixture was homogenized and centrifuged at 350× *g* for minutes. Again, the supernatant was decanted and the precipitate was washed with 10 mL of distilled water, homogenized and centrifuged. The supernatant was discarded and the final precipitate was mixed with 10 mL of zinc sulfate (density of 1.18) and centrifuged at 200× *g* for 1 min. The superficial material was collected with a Pasteur pipette and a drop was placed on a microscope slide and mixed with lugol solution for the identification of cysts.

Of the positive samples where cysts were identified, only those with a high quantity of cysts, more than 10 cysts per field (40× objective), were included for further analysis. Samples with fewer than 10 cysts per field (40× objective) did not successfully achieve PCR amplification. These were treated with a Sheather’s sucrose solution to purify and concentrate cysts [26] for posterior DNA extraction.

### 2.3. DNA Extraction

For DNA extraction, the first step was the lysis of cysts following the methodology proposed by Babaei et al. [27], with certain modifications, described next. From each concentrated sample, 200 µL were introduced in 2 mL cryovials with 200 µg of glass beads (0.1 mm diameter) and 500 µL of lysis buffer (100 mM NaCl, 50 mM Tris HCl, 100 mM EDTA, 1% SDS, pH 7.4), which was homogenized with a PowerLyzer 24 (Mo Bio Laboratories, Inc.) for 5 cycles of 2 min at 700 g and a resting period of 30 s between cycles. Following this, samples were put through a thermal shock treatment that consisted of 5 freeze–thaw cycles with liquid nitrogen and boiling water. Additionally, 40 µL of proteinase K (10 mg/mL) (Invitrogen, Carlsband, CA, USA) and 10 µL of SDS (1 M) were added; this solution was then incubated for 4 h at 55 °C. Finally, DNA extraction was performed by the phenol-chloroform method (CTAB method) described by Almeida et al. [28], with some modifications, described next. Each sample was added 400 µL of TE 1X buffer (100 mM tris-HCl pH 8, 10 mM EDTA) and 50 µL of lysozyme (10 mg/mL) (Sigma-Aldrich, Co., St. Louis, MO, USA), and homogenized and incubated for 1 h at 37 °C. Then, 100 µL of SDS (10%) and 10 µL of proteinase K (10 mg/mL) (Invitrogen, Carlsband, CA, USA) were added, and the mixture was homogenized and incubated for 30 min at 65 °C. This was followed by the addition of 100 µL of NaCl (5M) and 40 µL of pre-incubated CTAB (10%), and the mixture was vortexed until it turned milky; incubation for 30 min at 65 °C followed. Then, 400 µL of phenol–chloroform–isoamyl alcohol (24:24:1) were added and the solution was vortexed for 10 s and centrifuged at 13,200× *g* for 10 min. From this, 500 µL of the supernatant were transferred to a new vial and 500 µL of chloroform-isoamyl (24:1) were added; this was homogenized and centrifuged at 13,200× *g* for 10 min. Again, 500 µL of the supernatant were transferred to a new vial, adding 600 µL of absolute isopropyl alcohol. DNA was precipitated for 2 h at −20 °C and centrifuged at 13,200× *g* for 15 min. The supernatant was decanted and the DNA pellet was washed in 500 µL of ethanol (70%), centrifuged at 13,200× *g* for 20 s, followed by decanting of supernatant, and left to dry for 1 h at room temperature. Finally, the pellet was resuspended in 50 µL of ultrapure water and kept at −20 °C until further analysis.

### 2.4. Amplification of the β-Giardin Gene

For PCR amplification of the *β-Giardin* gene, the primers reported by Cacciò et al. [29] were used. Two rounds of amplification were performed, the first resulted in a 753 bp product with the primers G7 and G759, and the second produced a 384 bp fragment with the primers G759 and G376. PCR was performed with a final reaction volume of 20 µL, which contained 10 µL of GoTaq Green Master Mix 2X (Promega, Madison, WI, USA), 0.85 µL of each primer (10 µM), 6.3 µL of nuclease-free water and 2 µl of DNA (100 ng/µL). Cycle parameters for both rounds of PCR were as follows: initial denaturing cycle for 3 min at 95 °C, 35 denaturing cycles at 95 °C for 30 s each, an annealing cycle at 65 °C for 30 s, an extension cycle at 72 °C for 60 s, and a final extension cycle at 72 °C for 7 min. All runs included a positive and a negative control, the latter containing all of the elements of a normal reaction except for the DNA, which was substituted for nuclease-free water. DNA integrity and PCR products were analyzed through horizontal gel electrophoresis in agarose gel (1.5%) submerged in TAE 1× buffer (50 min, 80 volts), including a molecular weight marker of 100 bp (Promega, Madison, WI, USA) and Blue/Orange 6× loading buffer (Promega, Madison, WI, USA). Gels were posteriorly visualized with a UV digital photodocumentation system Gel Doc XR+ (Bio-Rad Laboratories) and the software Quantity One (Bio-Rad Laboratories).

### 2.5. Assemblage Typing

Typing of assemblages was performed through enzymatic digestion of the PCR products using the restriction enzymes *Hae III* and *Hha I* [29]. This produces, first, a banding pattern for typing all of the assemblages that can be found in dogs (A, B, C and D), and, secondly, the subtypes of assemblage A (AI and AII).

For the first round of digestion (the 753 bp fragment), the *Hae III* restriction enzyme (New England, BioLabs, Inc., Ipswich, MA, USA) was used, in a reaction volume of 10 µL, which contained 0.25 µL of enzyme, 1 µL of Cutsmart buffer (New England, BioLabs, Inc., Ipswich, MA, USA), 4.75 µL of nuclease-free water and 4 µL of the PCR product, incubated at 37 °C for 1 h.

For subtyping of assemblage A, which consists of the amplification of the second PCR product (384 bp), the restriction enzyme *Hha I* (Promega, Madison, WI, USA) was used. The reaction consisted of 10 µL of the second PCR reaction plus 0.25 µL of enzyme, incubated at 37 °C for 2 h.

Banding patterns were analyzed and observed through electrophoresis in agarose gel (3%) in TAE 1× buffer (120 min, 70 volts), and a molecular weight marker of 100 bp (Promega, Madison, WI, USA) and Blue/Orange 6× loading buffer (Promega, Madison, WI, USA). Gels were posteriorly visualized with a UV digital photodocumentation system Gel Doc XR+ (Bio-Rad Laboratories). Assemblage typing and subtyping was performed according to that described previously by Rodríguez et al. [30].

### 2.6. Sequencing

To confirm that PCR did amplify the targeted *β-Giardin* gene, the 384 bp fragment was sequenced using, in the first instance, the forward primer G376, and, in the second instance, using the reverse primer G759. Both primers were already reported by Cacciò et al. [29]. Sequencing was performed at the National Genomics for Biodiversity Laboratory (LANGEBIO–CINVESTAB, Irapuato, Guanajuato, Mexico). Sequencing files were analyzed with the software BioEdit version 7.0.5.3 [31], and a BLAST search was performed with the blastn suite in the NCBI Nucleotide database (https://blast.ncbi.nlm.nih.gov) for validation of the 384 bp fragment as the *β-Giardin* gene.

### 2.7. Statistical Analysis

To determine associations between the explicative variables and the presence of giardiasis, and estimate the risk factor (OR), a chi-squared analysis was performed using 2 × 2 contingency tables in the software EPIDAT 3.1 (https://www.sergas.es/Saude-publica/EPIDAT-3-1). The explicative variables were: age, origin (shelter, stray, domestic, and breeding establishment), gender and feces consistency.

## 3. Results

A total of 402 dog feces samples were analyzed: 112 from breeding establishments, 113 domestic, 102 stray, and 75 shelter. By gender, 222 were female and 180 male. Most corresponded to dogs older than six months, 89.8%. With regards to feces consistency, 276 animals presented firm feces and 126 were mounding feces.

### 3.1. Coproparasitoscopic Diagnosis

With regard to the coproparasitoscopic test, 102 samples resulted positive to the presence of *G. intestinalis* cysts, with a total prevalence of 25%. The frequencies obtained for age, feces consistency and gender are shown in Table 1, as well as the statistical significance for the association analysis between different risk factors for the presentation of disease, using a confidence interval of 95%. There was no significant association between the presence of the parasite and gender (*p* = 0.37); on the contrary, age did play an important role, as individuals younger than six months of age had twice the risk of having the parasite (OR = 2.01). In addition, the consistency of the feces was indicative of the presence of *G. intestinalis*, with a *p*-value of 0.0036, mounding feces proved to be more likely to be a related to the presence of the parasite.

Taking into account the type of dog population, or origin, the highest frequency of the parasite was for stray dogs (40.77%), followed by breeding establishment (26.21%), shelter (19.4%) and, finally, domestic (13.59%). In relation to these results, for the chi-squared, domestic dogs were considered as the reference group. Domestic dogs were the least associated to having the parasite (*p* < 0.05) (Table 2; Appendix B). On the other hand, the highest association was achieved by stray dogs (*p* = 0.0001), which also had the highest risk factor value (OR = 4.95). In second place were dogs from shelter (OR = 2.57) and following close were dogs from breeding establishment (OR = 2.24).

### 3.2. Amplification of the β-Giardin Gene and Assemblage Typing

Only the samples from the stray dog population achieved PCR amplification; these samples showed a higher number of cysts (more than 10 cysts per field with 40× objective). From these, 85.7% (*n* = 24) achieved successful amplification of both target DNA fragments (753 and 384 bp). One hundred percent (*n* = 24) belonged to assemblage A, of which 83.3% (*n* = 20) belonged to subtype AI and 16.7% (*n* = 4) were AII.

**Figure 1:**
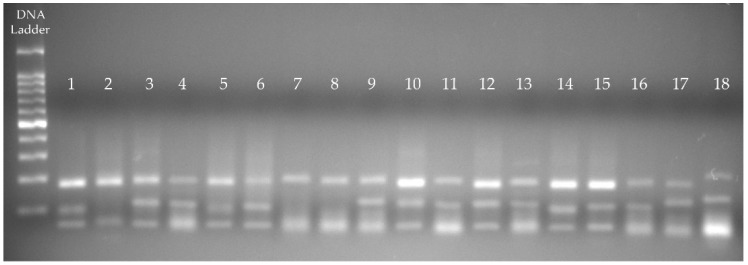
RFLP analysis of the 384 bp fragment (*G. intestinalis*) obtained from samples from dogs in the central region of Mexico. Lanes 1, 3–6, and 9–18 show the banding pattern that corresponds to subassemblage AI (190, 100 and 70 bp). Lanes 2, 7, and 8 show the banding pattern for subassemblage AII (210 and 70 bp).

In the following figure, we can see the result of the RFLP analysis and assemblage subtyping of the 384 bp fragment to 18 of the 24 amplified samples. Fifteen samples were genotyped as subassemblage AI, with bands at 190, 100 and 70 bp. Three samples (lanes 2, 7, and 8) were subtyped as AII, with bands at 210 and 70 bp (Figure 1).

### 3.3. Sequencing

Two sequences were retrieved from sequencing analysis, one for the forward primer G376 and another for the reverse primer G759 (Appendix A). Results from the BLAST search performed for these two sequences revealed a 100% and 94% identity value to the *β-Giardin* gene sequences reported in GenBank, respectively.

## 4. Discussion

The overall prevalence obtained for *G. intestinalis* in this study was 25%, which is higher in comparison to that reported globally, 15.2% [18], but similar to what has been previously reported for dogs of urban and rural areas in Sinaloa, which is located 1000 km northwest of Queretaro and is considered to be in the northern region of Mexico [32]. Even though Sinaloa presents hotter temperatures and higher humidity (26 °C annual average temperature/790 mm per m^2^ per year) than Queretaro (20 °C annual average temperature/554 mm per m^2^ per year), the prevalence was similar. Mexico City and Queretaro, on the other hand, are both within the central region of Mexico, located at a distance of 100 km from each other, and with very similar climatic conditions (18 °C/600 mm/m^2^ per year and 20 °C/554 mm/m^2^ per year [33], respectively). The lower prevalence found in our study in comparison to Mexico City (42%) may be due to the sampling method; while Ponce-Macotela et al. [19] obtained their samples directly from the intestines, where the parasite is always present, our sampling was from feces, and since the cysts are excreted rather intermittently [34], it is possible our prevalence is underestimated. A direct smearing method to detect trofozoites was not considered due to the sampling method used in this study, which consisted of collecting the feces from the ground, thus no diarrheic feces were obtained, which are considered to be the best for the recovery of trofozoites [35]. Furthermore, the direct smearing method has low sensitivity (34.7–55%) for detection of cysts due to the smaller amount of sample that is analyzed, in comparison to sedimentation/flotation techniques, which have a sensitivity of 65.2–83% [36].

The correlation analysis indicated that age is an important risk factor, it conveyed that young dogs (<6 months) are more likely to present the disease. This coincides with other studies performed previously [18,37,38,39]. In addition, as per type of dog population, stray dogs showed to be at an increased risk of having the parasite, in comparison to the other groups (breeding establishment, shelter and domestic); these data agree with reports from Brazil [39] and Bouzid et al. [18]. On the contrary, a study from Italy, by Capelli et al. [37], reported dogs from breeding establishments to be at greater risk. Furthermore, in Spain, prevalences for breeding establishments, shelter and domestic dogs were reported as 45.8%, 40.4% and 37.7%, respectively [40].

In relation to the results from DNA extraction and PCR, cyst lysis played a significant role: the better the cyst lysis is performed, the more DNA is available for extraction, therefore there is a higher possibility of obtaining amplification of the desired fragment through PCR. In this study, we achieved amplification from 85.7% of the samples, which represents a higher proportion than that achieved by Babaei et al. [27], where only 36% of the samples were apt for amplification. The different proportions obtained by these studies may be due to the methodology used for DNA extraction. Babaei et al. [27] used the traditional phenol-chloroform-isoamyl alcohol method, while in our study we performed an additional step with CTAB. This eliminates polymerase inhibitors that may be present in the feces and improves purity, though it may reduce DNA concentration [41]. This reduction in the concentration of DNA may explain why 14% (4/28) of the samples did not achieve amplification.

For the PCR, only the *β-Giardin* gene was targeted it has proven to be the most efficient for PCR amplification and for the differentiation of the assemblages. It presents high sensitivity for detection of infections by one or more assemblages, in comparison to the *18S rRNA* and *gdh* genes [42]. Furthermore, the *gdh* gene was not included for confirmation of results from *β-Giardin* because sensitivity for PCR amplification is lower [43,44]. Additionally, there have been studies that performed assemblage differentiation with both genes (*β-Giardin* y *gdh*) and found no significant differences in the results [30,45,46]. The only advantage for the *gdh* gene in those studies was that it was able to further differentiate subtypes of assemblage B [30,45], which was not present in our samples. Lastly, other authors performed amplification and sequencing of the different genes and obtained similar results across all genes [47,48,49], or with minor variations in one of the four genes used (*SSU rRNA*) [44]. However, the *SSU rRNA* gene, while it has high efficiency for the diagnosis of *Giardia*, it is not the best option for genotyping, unlike the *β-Giardin*, *tpi* y *gdh* genes [43]. The *tpi* gene, on the other hand, due to its variability, is a good phylogenetic marker for molecular evolution and epidemiological studies, and for the detection of mixed intragenotypic infections [50,51]. Due to the aforementioned, the gene of choice for this study met the necessary characteristics for accomplishing the proposed objective of this study.

Assemblage typing of the *G. intestinalis* positive samples indicated that all were type A. Because humans are susceptible to this assemblage, this highlights the risk of associated to zoonotic transmission [52]. Other studies have reported to only have found species-specific assemblages in dogs, which raises doubt for the risk to public health through zoonotic transmission [53,54]. However, in Río de Janeiro [46] and in the north of Mexico [21,32], the assemblages recovered from dogs were also type A.

Subtyping of the assemblages showed AI to be in higher proportion (83.3%) than AII (16.7%), which suggests that AI has higher preference for domestic animals [10,55]. In domestic dogs from Cuba [56], the prevalences for AI and AII were observed at 80% and 20%, respectively. In Sinaloa, Mexico, García-Cervantes et al. [32] reported a prevalence of 100% for AI, and Eligio-García et al. [21] reported 52.6% for AI and 47.4% for AII. However, the aforementioned studies included fewer animals, 5 and 19, respectively, than the present study (*n* = 24).

The distribution of the assemblages in dogs may be very influenced by environmental, social and cultural factors. Two possible cycles of transmission may exist: dog-to-dog transmission of species-specific assemblages and cross-infection between dogs and humans that involves the zoonotic assemblages (A and B) [10,19,42].

In Mexico, the stray dog population is problematic. Stray dogs inhabit rural and urban areas equally. In urban areas, stray dogs can easily be found in parks, where they usually defecate, disseminating parasites and microbes. Dog owners normally visit these parks to walk their dogs, usually letting them roam free for a while without a leash. As they roam freely, touching their snouts to the ground, domestic dogs are vulnerable to become infected with the parasites and microbes from stray dogs. This, in turn, makes domestic dogs a source of infection to their owners, which is where the risk to public health lies concerning this disease. On the other hand, dogs from shelters and breeding establishments more often are given preventive medicine care (deworming and deparasitizing treatments, vaccinations, medical examinations, etc.), so the risk of people in contact with them is reduced.

If we take into account the high frequency that we observed in this study with respect to *G. intestinalis* cysts in stray dogs, as well as the low dose required to infect another canine and cause the disease [6], this may suggest a high risk to public health due to environmental contamination, due to the cysts capacity to remain viable in water, food, and open grass-filled areas [5]. For this reason, parks are perfect sources of infection for domestic dogs and humans, as they are important recreational spots. Additionally, if 100% of the cysts excreted belong to assemblage A, the possibility of causing disease in humans is higher. Directly in relation to this, it is of utmost importance to control these types of infections (zoonosis) in dogs through preventive medicine programs aimed at this population. Furthermore, the efficiency of water treatment strategies needs to be improved, as this represents an important source of infection [6,57,58,59]. In addition, reducing this risk can be achieved through good hygiene practices education for people in order to improve the preparation of food and avoid consumption of contaminated products, which can be another important source of infection to humans [6,7].

## 5. Conclusions

The results of this study show that stray dogs in the central region of Mexico may be an important source of infection by *G. intestinalis* to other mammals, most importantly humans. The presence of assemblage A in these dogs poses a threat to public health through the contamination of water and outdoor recreational spaces in which both species coexist and interact. Control measures need to be implemented in order to reduce and eliminate this risk.

## Figures and Tables

**Table 1:** Sample distribution and statistical analysis for the association of risk factors (age, feces consistency and gender).

Factors		Total	Positive	Negative	Frequency	*p*-Value	OR	CI 95%
Age	<6 months	41	16	25	39.02	0.038 *	2.01	1.029–3.947
	>6 months	361	87	274	24.1			
Feces Consistency	Mounding	126	42	84	33.3	0.0036 *	2.009	1.251–3.226
	Firm	276	55	221	19.9			
Gender	Male	180	50	130	27.8	0.37	1.22	0.782–1.921
	Female	222	53	169	23.9			

* Statistically significant data (*p* < 0.05).

**Table 2:** Statistical analysis of association as per type of dog population (stray, shelter and breeding establishment; domestic dogs were considered as the reference group).

Dog population	Reference Group		
	*p*-Value	OR	CI 95%
**Stray**	0.0001 *	4.95	2.496–9.815
**Shelter**	0.0128 *	2.57	1.204–5.489
**Breeding Establishment**	0.028 *	2.24	1.107–4.557

* Statistically significant data (*p* < 0.05).

## Appendix A

### Appendix A.1. Sequencing Analysis for the Forward Primer G736

#### >B_Giardin_*G376*_Partial Sequence

CGTAAGGCGCACAGATGCATGGTGACCTCCTACGAGGACGTCGTCACGAAGATCCAGGGCGGCCTCACAGTGTATCGCAGCAGCGTGCCTCAGAAAGTCGCAGAGGGCTTCGCCCGCATCTCCGCCGCCATCGAGAAGGAGACGATCGCCCGCGAGAGGGCCGTCAGCGCTGCCACGACAGAGGCCCTCACAAACACGAAGCTCGTTGAGAAGTGTGTCAACGAGCAGCTCGAGAACGTCGCCTCGGAGATCCGCGCCATCCAGGAGGAGATCGACCGCGAGAAGGCAGAGCGCAAGGAGGCAGAGGACAAGATTGTTAACACACTCGAGGACGTCGTCTCGAAGATCCAGGGCGGCCTCACTGTTTCAGCTGAACAGGGTATGGGCGGGGTGGCAAAGGCGTGCTTCCCAAACAGCCAAAGAAAGTGAGAGATCCTCTCCTTAAGAATACATTTTATATGCTTCTACTGCCGTCAATTATACCCCGATGTTTTCAAAAAGACACCACAATGCCTCTATTGCTAACAGCGCAACCCTAGAACCTAAAGAAGTCGCTCTTCG

**Table A1:** BLASTn results for the sequences obtained by PCR amplification of the *G. intestinalis β-giardin* gene (Primer G376).

Description	Max Score	Total Score	Query Cover	E Value	Ident	Accession
Giardia intestinalis assemblage B isolate INI 49_53_57 beta giardin (bg) gene, partial cds	497	497	47%	10^−136^	100%	KX085488.1
Giardia intestinalis assemblage B isolate INI 21_68 beta giardin (bg) gene, partial cds	497	497	47%	10^−136^	100%	KX085486.1
Giardia intestinalis partial bg gene for beta giardin, isolate edr6	481	481	47%	10^−131^	98.8%	HG425171.1

### Appendix A.2. Sequencing Analysis for the Forward Primer G759

#### >B_Giardin_*G759*_Partial Sequence

TGAAGTGCGGCGCTGAGGATCTAGTCCGCTGCCTCCTTCCTGAGAGCCGCGATGGCGTCTTATGAGCGGGATGGCCTCAATCTGAAAGGCTTAGATCCCCAGCTGCTCCGTGACGCACTTCTCAACGAGCTTCGTGTTTGTGAGGGCCTCCGTCGTGGCAGCGCTGACGGCCCTCTCGCGGGCGATCGTCTCCTTCTCGATCGCGGCGGATATGCGGGCGAAGCCCTCTGCCACTTTCTCGTTGAGCTGGGCATACATCTTCTTCCTCTCGGCGGTCTCCGGGGCAATGCCTGTCTCGAGGTCGTTCAGGCTCTTGAGGGCCTCCTTCCTGAGAACCACTATGGCGCATTTATGTATTTCAGCCAAACACGTTGTGTGCGGCAGGGCGACAGCCTCTGTCCATAACAGCCATTTAGTGGGAAAGTTCGTGTGCTGAGACACACACTTTTTTTGCTTCTGGAGCATGTACTATACCGTCGTTATTTTAAAGACATAAAACCCTTCACCCCTATCGCTGAAATGGGTACCTTAGAAAATTAATCTTTCATCCTTCGAGCTATGGAGCCTAAAACAAGATAGCATAAGCCGTATCAGCATCAGCGACCGAACCGAGGAAGGGGACCCCCGCT

**Table A2:** BLASTn results for the sequences obtained by PCR amplification of the *G. intestinalis β-giardin* gene (Primer G759).

Description	Max Score	Total Score	Query Cover	E Value	Ident	Accession
Giardia intestinalis assemblage B isolate INI 49_53_57 beta giardin (bg) gene, partial cds	377	436	44%	2 × 10^−100^	94.3%	KX085488.1
Giardia intestinalis assemblage B isolate INI 21_68 beta giardin (bg) gene, partial cds	377	436	44%	2 × 10^−100^	94.3%	KX085486.1
Giardia intestinalis clone 10 beta giardin gene, partial cds	372	426	43%	10^−98^	93.9%	KR046097.1

## Appendix B

**Table A3:** Statistical analysis of association as per type of dog population (stray, shelter and domestic; breeding establishment dogs were considered as the reference group).

Dog Population	Reference Group		
	*p*-Value	OR	CI 95%
Stray	0.076	2.203	1.226–3.958
Shelter	0.6925	1.114	0.585–2.237
Domestic	0.028 *	0.445	0.219–0.903

* Statistically significant data (*p* < 0.05).

**Table A4:** Statistical analysis of association as per type of dog population (stray, breeding establishment and domestic; shelter dogs were considered as the reference group).

Dog Population	Reference Group		
	*p*-Value	OR	CI 95%
Stray	0.0455 *	1.925	1.008–3.672
Breeding Establishment	0.6925	0.873	0.446–1.707
Domestic	0.0128 *	0.3888	0.182–0.830

* Statistically significant data (*p* < 0.05).

**Table A5:** Statistical analysis of association as per type of dog population (shelter, breeding establishment and domestic; stray dogs were considered as the reference group).

Dog Population	Reference Group		
	*p*-Value	OR	CI 95%
Shelter	0.0455 *	0.519	0.272–0.991
Breeding Establishment	0.0076 *	0.453	0.252–0.815
Domestic	0.0001*	0.202	0.101–0.400

* Statistically significant data (*p* < 0.05).
